# Case report: Stereotactic body radiation therapy with 12 Gy for silencing refractory ventricular tachycardia

**DOI:** 10.3389/fcvm.2022.973105

**Published:** 2022-11-03

**Authors:** Shan-Hui Huang, Yen-Wen Wu, Pei-Wei Shueng, Shan-Ying Wang, Meng-Chieh Tsai, Yuan-Hung Liu, Wen-Po Chuang, Heng-Hsu Lin, Hui-Ju Tien, Hsin-Pei Yeh, Chen-Hsi Hsieh

**Affiliations:** ^1^Division of Cardiology, Cardiovascular Medical Center, Far Eastern Memorial Hospital, New Taipei City, Taiwan; ^2^School of Medicine, National Yang Ming Chiao Tung University, Taipei City, Taiwan; ^3^Department of Nuclear Medicine Center, Far Eastern Memorial Hospital, New Taipei City, Taiwan; ^4^Division of Radiation Oncology, Department of Radiology, Far Eastern Memorial Hospital, New Taipei City, Taiwan; ^5^Department of Biomedical Imaging and Radiological Sciences, National Yang Ming Chiao Tung University, Taipei City, Taiwan; ^6^Division of Radiology, Department of Radiology, Far Eastern Memorial Hospital, New Taipei City, Taiwan; ^7^Department of Electronic Engineering, Asia Eastern University of Science and Technology, New Taipei City, Taiwan; ^8^School of Medicine, Institute of Traditional Medicine, National Yang Ming Chiao Tung University, Taipei City, Taiwan; ^9^Head and Neck Cancer Surveillance and Research Group, Far Eastern Memorial Hospital, New Taipei City, Taiwan

**Keywords:** refractory, ablative, radiosurgery, stereotactic body radiation therapy, ventricular tachycardia, volumetric-modulated arc therapy

## Abstract

**Background:**

Encouraging results have been reported for the treatment of ventricular tachycardia (VT) with stereotactic body radiation therapy (SBRT) with 25 Gy. SBRT with 12 Gy for refractory VT was designed to reduce long-term cardiac toxicity.

**Methods:**

Stereotactic body radiation therapy-VT simulation, planning, and treatment were performed using standard techniques. A patient was treated with a marginal dose of 12 Gy in a single fraction to the planning target volume (PTV). The goal was for at least ≥ 95% of the PTV to be covered by at least 95% of 12 Gy radiation.

**Results:**

From April 2021 through June 2022, a patient with refractory VT underwent treatment. The volume for PTV was 65.8 cm^3^. The mean radiation dose administered to the heart (the heart volume excluding the PTV) was 2.2 Gy. No acute or late toxicity was observed after SBRT. Six months after SBRT, the patient experienced new monomorphic right ventricular outflow tract (RVOT) VT. Interestingly, the substrate of the left ventricular basal to middle posteroseptal wall before SBRT was turned into scar zones with a local voltage < 0.5 mV. Catheter ablation to treat RVOT VT was performed, and the situation remains stable to date.

**Conclusion:**

This study reports the first patient with refractory VT successfully treated with 12.0 Gy SBRT, suggesting that 12 Gy is a potential dose to treat refractory VT. Further investigations and enrollment of more patients are warranted to assess the long-term efficacy and side effects of this treatment.

## Introduction

Ventricular tachycardia (VT) is a life-threatening disease that is caused by electric reentry within and around patches of myocardial scarring (1), especially in patients with postmyocardial infarction (post-MI) heart failure. The implantation of a cardioverter-defibrillator (ICD) prevents sudden cardiac death, but ICD shocks are also associated with mortality (2, 3). Recently, cardiac imaging and electroanatomic mapping (EAM) have improved in guiding the ablation procedure to identify the VT substrate and to ablate these regions, with encouraging results (4, 5). However, following such procedures, the recurrence rate after a first VT ablation is 12.0–62.0% (6, 7), so patients must undergo repeated catheter ablation procedures (8).

Stereotactic body radiation therapy (SBRT) is an advanced external beam radiotherapy technique that precisely delivers a high radiation dose with an image-guided technique to targets in a few fractions (9, 10). Recently, encouraging results were reported for patients with VT who were treated with SBRT (11, 12). Cuculich et al. (11) documented that all awake patients treated with SBRT in a single fraction of 25 Gy exhibited a 99.9% decrease in the VT burden compared to the baseline. In a subsequent phase I/II trial, the frequency of VT episodes or premature ventricular contraction (PVC) burden was reduced by 75.0% in 89.0% of patients, as reported by Robinson et al. (12).

However, 10.5% of patients in that study experienced grade 3 treatment-related adverse effects, 11.1% developed grade 2 radiation pneumonitis, and 28% exhibited pericardial effusions (12). Calculation of the late radiation response of the heart by equivalent dose in 2-Gy fractions (EQD2, corrected for fractionation with alpha/beta ratio = 2.5) showed that the administration of 25 Gy in one fraction might actually elicit the same response as 152 Gy (13). Interestingly, radiosurgery with 12 Gy provides a high rate of control of acoustic neuroma (14), which provides insight into abnormal nerve behavior that may be reversed by radiosurgery with marginal doses of 12 Gy.

A stereotactic ablative radiosurgery (SARS) for refractory VT (SARS-VT) trial was established at our institution after obtaining approval from the Human Experimentation Committee of Far Eastern Memorial Hospital (FEMH108074-F) to reduce long-term cardiac toxicity, improve quality of life, and search for the optimal radiation ablation dose. Here, we report the first patient in the world with refractory VT who was treated with SBRT using 12 Gy radiation and was followed for more than 1 year. This patient had a favorable outcome with a satisfactory quality of life.

## Case report

### Patient history

A 63-year-old man had a past history of type 2 diabetes mellitus, hypertension, hyperlipidemia, chronic kidney disease and congenital bicuspid aortic valve stenosis with coronary artery disease. Coronary artery angioplasty and stenting to the right coronary artery and left circumflex artery had been performed more than 10 years prior. The patient had developed congestive heart failure (New York Heart Association, functional class II to III) with a reduced ejection fraction and a left ventricular ejection fraction (LVEF) of less than 35% in the past 3 years. Two years before this presentation, he received xenograft aortic valve replacement due to rapidly progressive aortic valve stenosis and coronary artery bypass graft with saphenous vein graft (SVG) anastomosis to the left anterior descending artery. A dual-chamber implanted cardioverter-defibrillator (ICD) was also implanted for subsequent sustained monomorphic VT.

**FIGURE 1:**
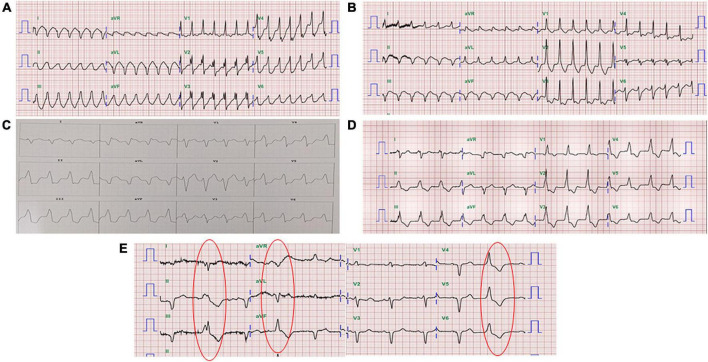
Twelve-lead electrocardiography of the patient. **(A)** VT with right bundle branch block morphology and an inferior axis. **(B)** VT with right bundle branch block morphology and a superior axis. **(C)** VT with the origin in the RVOT. **(D)** Slow VT with all positive concordance in the precordial leads and an inferior axis suggested at a different location from the initial therapy zone. **(E)** The morphology of ventricular premature beats was the same as the morphology of the slow VT.

**FIGURE 2:**
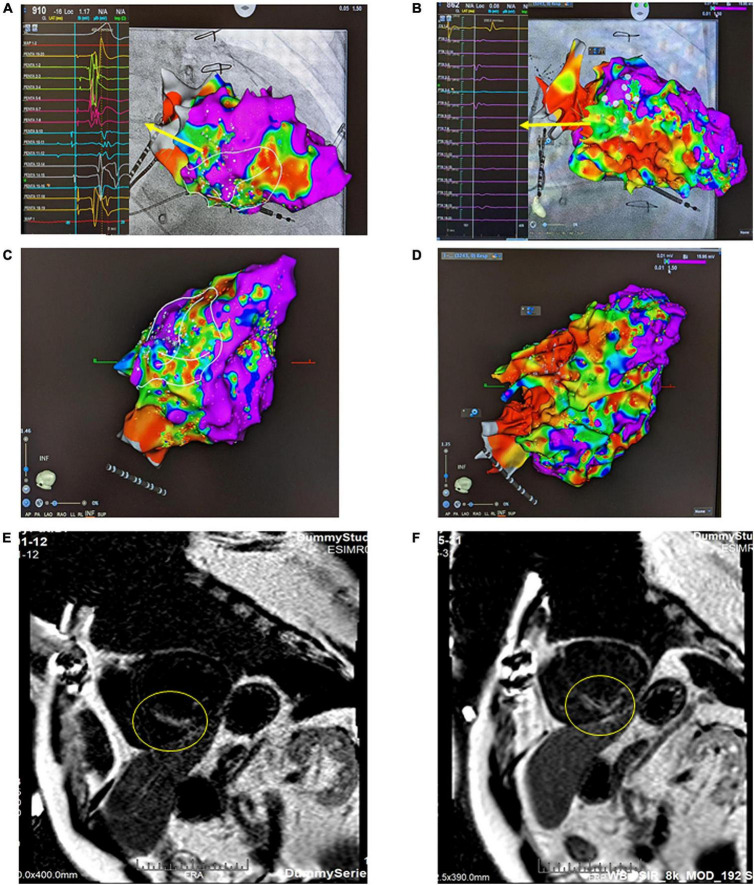
Bipolar voltage mapping with a 3D CARTO mapping system (Biosense, Diamond Bar, CA). **(A)** The presence of a large, uneven, mixed scar and viable zone in the area of the left ventricular basal to the middle posteroseptal wall from the right anterior oblique (RAO) view. The arrow indicates LAVA, the substrate, as the target ablation zone. **(B)** After SBRT, the therapy zone became a scar zone. The arrow indicates local voltage < 0.5 mV. **(C)** Inferior view of the left ventricle (LV) before SARS. **(D)** Inferior view of the left ventricle (LV) after SBRT. **(E)** Cardiac MRI using a wide-band sequence before SBRT. A delay in the enhancement of the mid-basal septal wall and mid-inferior wall was noted, which indicated scar formation in this area. **(F)** Cardiac MRI after SBRT. The previously shown delay in enhancement of the mid-basal septal wall and mid inferior wall was still present; however, the affected area was slightly larger than that on the previous MRI.

One year ago, the patient had a recurrent VT storm with ICD shock. The twelve-lead electrocardiogram revealed two different morphologies of clinical VT (Figures 1A,B). Both VT morphologies indicated right bundle branch block morphology; one involved the superior axis, and the other involved the inferior axis, which suggested an origin of VT from the left ventricular septal wall. During the electrophysiology study, bipolar voltage mapping with a three-dimensional (3D) CARTO mapping system (Biosense, Diamond Bar, CA) suggested the presence of a large, uneven, mixed scar and viable zone in the area of the left ventricular basal to middle posteroseptal wall (Figures 2A,C,E). We defined the border zone as where the voltage was <1.5 mV. Local abnormal ventricular activity (LAVA) was identified as a substrate using a PENTARAY^®^ NAV ECO high-density mapping catheter near the scar margin. Endocardial ablation with LAVA elimination was performed with an acute end point of non-inducibility under ventricular extrastimuli. However, recurrent VT storms still occurred 5 months after catheter ablation, with a total of 21 episodes of ICD therapy (15 events of antitachycardia pacing and 6 events of shock). The patient received a detailed explanation of the treatment that would be given in the SARS-VT trial from both the treating electrophysiologist (the first author) and the radiation oncologist (the last author). After discussion, he agreed to receive ablated cardiac radiotherapy at a dose of 12 Gy. The patient provided written informed consent to receive treatment. All the authors participated in data collection and analysis.

### Stereotactic body radiation therapy planning and delivery

**FIGURE 3:**
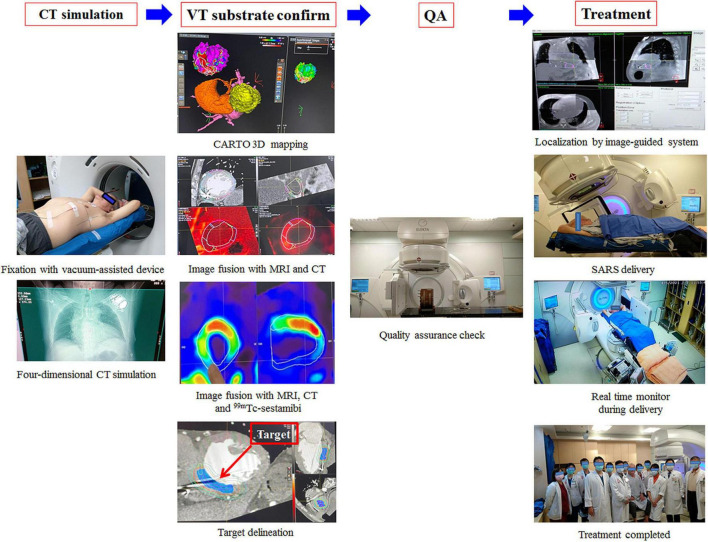
Multimodal imaging combined with scar imaging, including the CARTO mapping system (Biosense, Diamond Bar, CA), CT, MRI and ^99^*^m^*Tc-sestamibi MPI, was used offline to delineate a target for ablation. A plan was developed in the Pinnacle3 planning system (version 9.8.1, Philips Medical Systems, Madison, WI, USA). One day before treatment, quality assurance was performed. On the day of treatment, the patient was immobilized on the vacuum device, and cone-beam computed tomography (CBCT) was used for patient setup and target localization. The treatment unit was aligned with the patient, and a dose of 12 Gy was delivered precisely by volumetric-modulated arc therapy (VMAT) through a linear accelerator machine (Versa HDTM, Elekta, Crawley, West Sussex, UK) with 6 MV photons.

The procedural workflow is shown in Figure 3. Multimodal imaging combining scar imaging, including the 3D CARTO mapping system (Biosense, Diamond Bar, CA), respiratory phase-correlated 4-dimensional computed tomography (4D-CT) simulation with a 2.5 mm slice thickness (Discovery CT590 RT, GE Healthcare, Chicago, IL, USA), cardiac magnetic resonance imaging (MRI) and ^99^*^m^*Tc-sestamibi myocardial perfusion imaging was done offline to delineate a target for ablation. The substrate was targeted at the clinical target volume (CTV) and was segmented through corroboration of all previously acquired imaging and electrocardiographic imaging data by the radiation oncologist and electrophysiologist. An internal target volume (ITV) was created from the phased-binned 4D-CT to account for the maximum range of motion that was assessed by reviewing the playback of all the phases of the 4D-CT overlaid with the reference CT scan. The amplitude for the target was approximately 5 mm. This result was similar to previous reports (12, 15, 16). The data reported that cardiac motion was variable depending on the specific substructure of the heart but was within 5 mm (15, 16). The planning target volume (PTV) was generated as a 3-mm volumetric expansion from the ITV. A plan was developed in the Pinnacle3 planning system (version 9.8.1, Philips Medical Systems, Madison, WI, USA).

**FIGURE 4:**
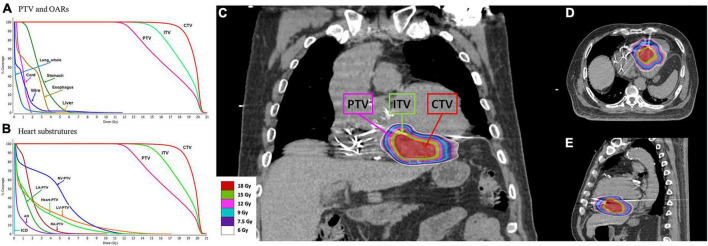
The substrate was targeted at the clinical target volume (CTV). An internal target volume (ITV) was created from the phased-binned 4D CT images to account for the maximum range of motion that was assessed by reviewing the playback of all the phases of the 4D-CT overlaid with the reference CT scan. The planning target volume (PTV) was generated as a 3-mm volumetric expansion from the ITV. The goal was for at least ≥ 95% of the PTV to be covered by at least 95% of the prescribed dose (12 Gy). Optimization was performed to place prescription hotspots (areas receiving > 100% of the prescribed dose) within the ITV and CTV, enabling the ITV and CTV to be covered by up to 125% and 150% of the prescribed dose, respectively. **(A)** Dose volume histogram (DVH) for the PTV and organs at risk. **(B)** DVH for the PTV and heart substructures. RV, right ventricle; LV, left ventricle; RA, right atrium; LA, left atrium; AO, aorta; ICD, cardioverter-defibrillator. **(C)** Coronal view of the plan with color wash. **(D)** Transverse view of the plan with color wash. **(E)** Sagittal view of the plan with color wash.

A dose of 12 Gy in a single fraction was prescribed to the PTV with the goal of achieving maximal dose coverage while avoiding a dose in excess of the calculated dose constraints for surrounding organs, including the heart and coronary arteries. The goal was for at least ≥ 95% of the PTV to be covered by at least 95% of the prescribed dose (12 Gy). Beam geometry was optimized to avoid the ICD geometrically. Optimization was used to place prescription hotspots (areas receiving > 100% of the prescription dose) within the ITV and CTV, enabling the ITV and CTV to be covered by up to 125 and 150% of the prescribed dose, respectively (Figure 4).

The criteria for the arteries, esophagus, stomach, lungs, and spinal cord were based on those used in a previous report (16). The plan was subjected to and passed standard internal physics quality assurance on a calibrated phantom 1 day before delivery. On the day of treatment, the patient was immobilized on the vacuum device (Klarity, R7515NL-44NL). We used cone-beam computed tomography (CBCT) for patient setup and target localization. The treatment unit was aligned with the patient, and SBRT of 12 Gy was delivered precisely without sedation or anesthesia by an image-guided radiotherapy-equipped linear accelerator (volumetric-modulated arc therapy, VMAT, Versa HDTM, Elekta, Crawley, West Sussex, UK).

## Results

### Patient and treatment

The CTV, ITV, and PTV were recorded. The target volumes for the CTV, ITV, and PTV were 23.23, 42.45, and 65.75 cm^3^, respectively. The conformal index was 1.03. The treatment dose rate was 1,400 MU/min for a total marginal dose of 12 Gy with 6 MV flattening-filter-free beams, and the duration of the treatment session was 24 min. The mean radiation dose administered to the heart (the heart volume excluding the PTV) was 2.2 Gy. The maximal doses (<0.03 cc) administered to the left and right coronary arteries were 4.2 and 6.9 Gy, respectively. The maximal dose administered to the mitral valve was 6.5 Gy. The medium doses to the left and right ventricles were 0.8 and 5.0 Gy, respectively. The maximal dose administered to the spinal cord and maximal esophageal dose were 2.0 and 4.4 Gy, respectively. The dosimetry details are shown in Table 1.

**TABLE 1:** The calculated doses to organs at risk and heart substructures for patients treated with stereotactic body radiation therapy (SBRT) 12 Gy.

Prescription to PTV	SBRT (Gy)	EQD2 (Gy, α/β = 10)	EQD2 (Gy, α/β = 2.5)	SBRT (Gy, FEMH)	EQD2 (Gy, α/β = 10)	EQD2 (Gy, α/β = 2.5)	Decrease (%) when compared to 25.0 Gy (α/β = 10)	Decrease (%) when compared to 25.0 Gy (α/β = 2.5)
	25.0	72.9	152.8	12.0	22.0	38.7		
**Organs at risk**							48.7–93.0%	55.0–96.3%
Spinal cord	Dmax ≤ 14 (16, 20, 29, 33)	28.0	51.3	Dmax: 2.0	1.9	1.9	93.0%	96.3%
Esophagus	Dmax ≤ 14.5–19.0 (16, 20, 29, 33, 49)	29.6–45.9	54.8–90.8	Dmax: 4.4	5.3	6.8	82.1–88.5%	87.6–92.5%
Stomach	Dmax < 12.4–22.0 (20, 29)	23.2–58.7	41.1–119.8	Dmax: 8.0	11.9	18.5	48.7–79.7%	55.0–84.6%
Total lung	V7 Gy remaining volume > 1500 c.c. (16, 20, 29, 33)	V9.9	V14.8	V7 Gy remaining volume:2319.8 c.c.	V9.9	V14.8		
Liver	V11.0 remaining volume > 700 c.c. (16)	V19.3	V33.0	V11.0 remaining volume:1579.09 c.c.	V19.3	V33.0		
**Heart substructures**							51.2–91.8%	58.3–98.3%
Mean Heart	6.0 (20)	8.0	11.3	2.2	2.3	2.3	71.3%	79.6%
Aorta	Dmax: 20.0–25.0 (49)	50.0–72.9	100–152.8	Dmax: 7.2	10.4	15.6	79.2–85.7%	84.4–89.8%
Left atrium	Median dose ≤ 4.4 (49)	5.3	6.8	Median dose: 0.9	0.8	0.6	85.5%	91.2%
Right atrium	Median dose ≤ 4.4 (49)	5.3	6.8	Median dose: 1.8	1.7	1.7	67.9%	75.0%
Left anterior descending artery	Dmax: 14.0–22.0 (20, 29, 49)	28.0–58.7	51.3–119.8	Dmax: 4.2	4.8	6.3	82.9–91.8%	87.8–94.7%
Right coronary artery	Dmax: 12.0–22.0 (20, 29, 49)	28.0–58.7	38.7–119.8	Dmax: 6.9	9.8	14.5	65.0–83.3%	62.5–87.9%
Left ventricle	Medium: 11.3 (16)	20.1	34.7	Medium: 0.8	0.7	0.6	96.5%	98.3%
Right ventricle	Medium: 8.3 (16)	12.7	19.9	Medium: 5.0	6.2	8.3	51.2%	58.3%
Aortic valve	Median dose: 3.5 (16)	3.9	4.7	Median dose: 1.1	1.1	0.9	73.7%	80.9%
Mitral valve	Dmax: < 20.0 (29)	50.0	100.0	Dmax: 6.5	8.9	12.9	82.2%	87.1%
ICD (major electronics)	Dmax ≤ 2.0 (49)	2.0	2.0	Dmax: 0.03	0.03	0.02	98.5%	98.5%

The data were compared to the constraints or dose limitations for patients who were treated with SBRT 25 Gy.

### Safety

No acute toxicity was observed during or immediately after SBRT. No adverse effects were observed from ICD during or after SBRT. No adverse events were related to SBRT during the whole 1-year observation period.

### Efficacy

**FIGURE 5:**
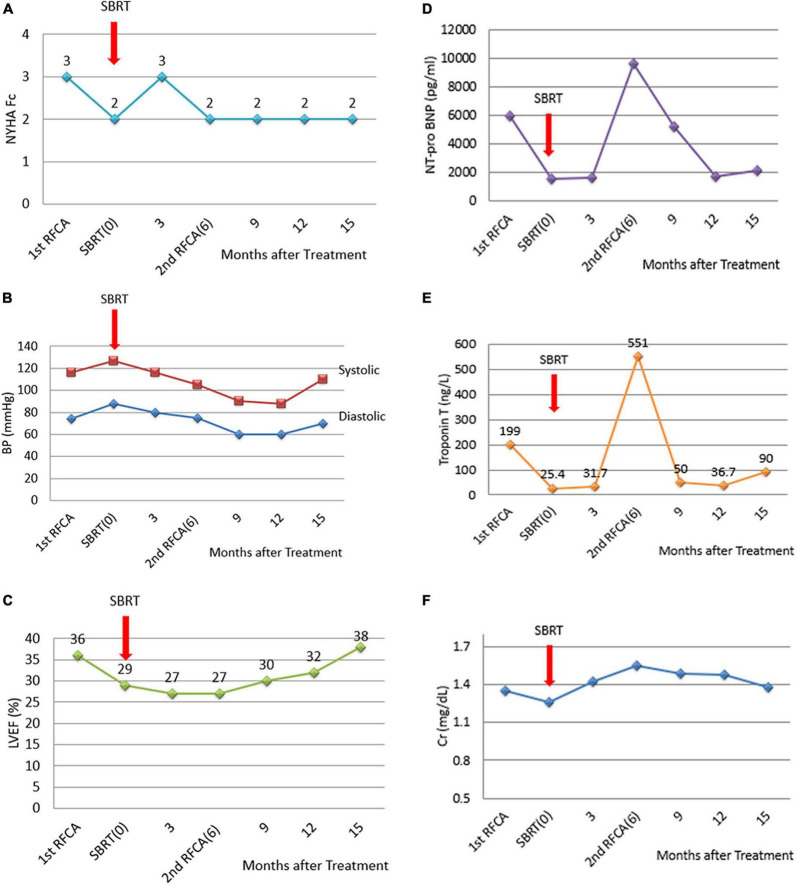
Diagrams of the NYHA functional classification, blood pressure, LVEF and laboratory data with a timeline for the patient before, during and after the treatment. **(A)** NYHA functional classification, explaining the severity of heart failure. **(B)** Blood pressure. **(C)** LVEF improved slightly after catheter ablation to the RVOT VT. The second electrophysiological study documented successful local treatment with SBRT using 12 Gy at the left ventricular basal to middle posterior septal wall. However, **(D)** NT-pro-BNP and **(E)** Troponin T levels increased 6 months after SBRT because of recurrent electric storms derived from the RVOT VT. After catheter ablation of the RVOT VT, serum NT-proBNP and troponin T levels decreased. **(F)** Serum creatinine levels remained unchanged.

Before SBRT, the patient received two types of antiarrhythmic agents (amiodarone and mexiletine), a maximal tolerated dose of bisoprolol, sacubitril/valsartan and empagliflozin (25 mg daily), which have been suggested to reduce the combined risk of cardiovascular death or hospitalization for heart failure in patients with heart failure. After SBRT and the second electrophysiology study, antiarrhythmic agents and optimal medical treatments for heart failure remained unchanged. Six months after SBRT, 16 episodes of antitachycardia pacing (ATP) plus ICD shock occurred in 24 h. Based on the protocol, a second SBRT needed to be delivered. The patient underwent a second electrophysiology study to confirm the original therapy zone. Interestingly, the substrate of the left ventricular basal to middle posteroseptal wall before SBRT had turned into scar zones with a local voltage < 0.5 mV (Figures 2B,D,F). One monomorphic right ventricular outflow tract (RVOT) VT was easily inducible (Figure 1C). No low-voltage zone or LAVA was observed in this zone through high-density voltage mapping. Catheter ablation to the RVOT VT was performed with an acute end point of non-inducibility under ventricular extrastimuli. During follow-up, another morphology of sustained slow VT with a rate of 92 bpm was noted, without hemodynamic compromise. The morphology of slow VT was all positively concordant in the precordial leads and inferior axis, which indicated a different origin of VT (Figure 1D). Due to the wider QRS and electric replacement interval of the ICD, we upgraded the ICD to a cardiac resynchronization therapy defibrillator (CRT-D). The upgrade to CRT-D may also significantly reduce the frequency of VT. The burden of ventricular arrhythmia has been markedly reduced since then. The morphology of the ventricular premature complex was the same as that of slow VT (Figure 1E). Figure 5 describes the New York Heart Association (NYHA) functional classification, blood pressure, left ventricular ejection fraction (LVEF) and laboratory data with a timeline for the patient before, during and after treatment.

## Discussion

This report is the first to describe SBRT with a PTV dose of 12 Gy performed in a patient with recurrent VT. The target volume for SBRT was delineated using the CARTO mapping system, with subsequent matching by MRI, ^99^*^m^*Tc-sestamibi myocardial perfusion imaging and 4D-CT imaging. SBRT with 12 Gy silenced the abnormal electrical activity of the substrate and successfully reduced the VT burden during the follow-up period.

Cardiac SBRT with 25 Gy in one fraction is a new and innovative treatment for patients with refractory VT (Table 2). To date, eight case reports (17–24) and 11 case series (11, 12, 25–32) have been published. Ninety-one patients were treated with a radiation dose of 25 Gy, and one patient was treated with 24 Gy. The rate of reduction in the VT burden by SBRT was approximately 69–90% (11, 12, 26, 27, 31). However, the rate of recurrent VT after SBRT was 20–100%. Treatment with 25 Gy in one fraction, when corrected for the late radiation effect for fractionation with alpha/beta ratio = 2.5, was equivalent to 152 Gy (13). During the ENCORE VT study (12), two patients experienced grade 3 heart failure exacerbation and pericarditis. Additionally, five patients presented with grade 2 pericardial effusions, and two patients experienced grade 2 radiation pneumonitis. Similarly, Neuwirth et al. (26) reported that one patient exhibited grade 3 late radiation-related progression of mitral regurgitation and changes in valvular morphology 17 months after radiosurgery. Qian et al. (33) reported that 3 of six patients experienced adverse effects after SBRT with 25 Gy: one suffered from pneumonia 1 month later; another had severe left ventricular dysfunction, mitral regurgitation and tricuspid regurgitation and developed heart failure decompensation 104 days later; and the other presented with moderate pericardial effusion 396 days later. Therefore, the potential risks for late radiotherapy-related effects of cardiac toxicity should still be considered with caution.

**TABLE 2:** Comparison of a patient with refractory VT treated with SBRT using a dose of 12 Gy at the Far Eastern Memorial Hospital (FEMH) with a selected published series of patients treated with SBRT.

References	Modality	PTV volume (c.c.)	Number of patients	Dose (Gy)	Treatment time	Episodes before RT	Episodes after RT	VT recurrence	Follow-up (mo: month; wk: week; d: day)
Loo et al. (17)	CyberKnife	NR	1	25	90 min	VT: 562/2 mo	VT: 52/mo	3 mo post-SBRT 100% (1/1)	9 mo
Cvek et al. (18)	CyberKnife	NR	1	25	114 min	PVCs: 9–10%	PVCs: 1–3%	NR	120 d
Wang et al. (25)	CyberKnife, TrueBeam Linear Accelerator	NR	4	25	Mean: 64.2 min	NR	NR	NR	NR
Cuculich et al. (11)	TrueBeam Linear Accelerator	49	5	25	14 min	Mean VT: 1315	Mean VT: 4	4 wk after SBRT, 20% (1/5, Patient 4) of patients had recurrent VT	12 mo
Haskova et al. (19)	CyberKnife	NR	1	25	NR	Incessant VT	Disappeared	NR	8 mo
Jumeau et al. (20)	CyberKnife	21	1	25	45 min	LVEF: 15%	LVEF: 30%	NR	4 mo
Robinson et al. (12)	TrueBeam or Edge Linear Accelerator	Median: 98.9	19	25	Median: 15.3 min (5.4–32.3)	119	3	69% (11/16) of patients had recurrent VT between the end of the 6-wk blank period and the 6-mo follow-up.	13 mo
Neuwirth et al. (26)	CyberKnife	22.15	10	25	Mean: 68 min	212	26	In total, 80% (8/10) of patients had recurrent VT. (1) Patients 4 and 1 experienced recurrences of VT up to 3 and 6 months after radiosurgery, respectively. (2) Patients 6 and 8 presented with a higher number of VT episodes after SBRT. (3) Three of the 10 patients experienced an electric storm.	Median: 28 mo
Krug et al. (21)	TrueBeam Linear Accelerator	42.2	1	25	55 min	VT: 5.1	VT: 1.6	One more week 100% (1/1)	57 d
Zeng et al. (22)	CyberKnife	NR	1	24	NR	VT: 189	VT: 0	NR	4 mo
Lloyd et al. (27)	TrueBeam linear accelerators	81.4	10	25	30 min	VT: 1065	VT: 332	Recurrent but with a 94% reduction in VT seconds	176 d
Gianni et al. (28)	CyberKnife	143	5	25	82 min	Total VT: 296	Total VT: 306	All patients (100%)	Mean: 12 mo
Martí-Almor et al. (23)	TrueBeam Linear Accelerator	–	1	25	–	Right ventricular ejection fraction: 30%	Right ventricular ejection fraction: 33%	NR	4 mo
Mayinger et al. (24)	MR-Linac with 6 MV flattening-filter-free photons	269	1	25	2 h and 28 min	–	VT aggravation 24 h after SBRT	NR	3 mo
Ho et al. (29)	TrueBeam Linear Accelerator	Mean: 54.5	7	25	Median beam-on time: 12.7 min	88	23	Excluding 1 patient who died, 50% (3/6: Patients 1, 2, and 5) of patients had recurrent VT.	Median: 14.5 mo
Yugo et al. (30)	TrueBeam Linear Accelerator	83.2	3	25	Mean: 71 min	140	54	All patients (100%)	NR
Lee et al. (31)	Linear Accelerator (Varian Medical Systems/Elekta)	94.5	7	25	Mean: 38.7 min	NR	The overall reduction in the VT burden was 85%	Recurrent VT but no clear state	6 mo
Chin et al. (32)	Novalis Tx Medical Linear Accelerator	121.4	8	Median: 25	average: 18.2 min	Median VT: 35	Median VT: 10.5	Excluding 4 patients who died, 100% (4/4) of the patients had recurrent VT after SBRT. (1) Patient 2: at 1 year (2) Patient 3: at 8 mo (3) Patient 6: at 3 mo (4) Patient 7: at 1 mo	7.8 mo
Qian et al. (33)	TrueBeam Linear Accelerator	319.5	6	25	Beam-on time: 13.8 min	Median VT: 42	Median VT: 29	In total, 83% (5/6) of the patients had recurrent device-treated VT at 97 d.	231 d
FEMH	VMAT Linear Accelerator (Versa HD)	65.75	1	12	24 min			No recurrence	16 mo

D, days; min, minutes; mo, months; NR, not reported; wk, weeks; VMAT, volumetric-modulated arc therapy.

SBRT (20 Gy or higher) is associated with reduced capillary density, myocardial degeneration and fibrosis (34, 35). Endothelial vacuolization and disruption of gap junctions on electron microscopy specimens is noted after SBRT as relatively acute effects that occur well before cellular DNA damage–related apoptosis and fibrosis begin (27). Additionally, severe architectural disruption, inflammatory cell infiltration, fibrin deposition and necrosis in myocardial tissue caused by high-dose irradiation in swine have been reported (22, 30, 31). Notably, irradiation with 16 Gy to the mouse heart induced mitochondrial damage and metabolic alterations of the cardiac tissue and led to structural remodeling, functional injury and fibrotic alterations (36). Interestingly, the severity and extent of myocardial injury, such that it reduced resting myocardial perfusion in rats, became more evident at the end of 6 months after 20 Gy heart irradiation in a single fraction (37). Similarly, when ^13^N-ammonia PET/CT myocardial perfusion imaging (MPI) was used to detect changes in myocardial perfusion induced by the administration of 20 Gy in one fraction to beagle dogs, the data showed a 10% reduction in LVEF at 6 and 12 months after irradiation (38). Similar findings have also been recently documented in human myocardium after SBRT, consistent with the preclinical models (19). Considering all these published observations, high-dose irradiation of the heart potentially results in cardiac damage.

More importantly, the published data show that the potential rate of recurrent VT after SBRT is 20–100% (Table 1). In contrast to those who received 25 Gy, three patients who received lower SBRT doses (median dose of 20 Gy) achieved clinically positive outcomes, as reported by Qian et al. (33). Ho et al. (29) delivered 20 Gy to the PTV with effective results that were lower than those of other reports. Lehmann et al. (39) suggested that ablated RT doses to create a complete atrioventricular conduction block and point doses to the coronary arteries should be < 10 Gy to remain safe. Radiosurgery with 12 Gy provides a high control rate for acoustic neuromas (14). Additionally, for patients with benign tumors adjacent to the anterior visual pathway who were treated by single-fraction stereotactic radiosurgery with less than 12 Gy vs. 12 Gy, the probability of radiation-induced optic neuropathy was 0% vs. 10% (40). Sharma et al. (41) demonstrated that 12 Gy caused mitochondrial degeneration. Mitochondria play an important role in cellular stress signals activated for acute, chronic nerve cell injury and nerve cell death (42) and maintain homeostasis and cellular integrity (43). Dysfunctional mitochondria affect neuronal trafficking across neurons. As mentioned above, mitochondrial damage in cardiac tissue could lead to structural remodeling, functional injury and fibrotic alterations (36). Moreover, 12 Gy irradiation has upregulated matrix metalloproteinase (MMP)-2 and MMP-9 activity. MMP-2 and MMP-9 degrade collagen IV of basement membranes, weakening the structural integrity (44). Notably, in the study of the late radiation response of canines, the median effective dose (ED_50_) for pericardial fibrosis in 2 Gy fractions was 46.1 Gy (13). However, for 12 Gy in one fraction corrected for fractionation with alpha/beta ratio = 2.5, the EQD2 was 38.7 Gy. Therefore, abnormal nerve reentry may be silenced by degenerating or dysfunctional mitochondria related to SBRT of 12 Gy, which could affect the neuronal trafficking process, structural remodeling and cellular integrity but also decrease the risk of pericardial fibrosis.

The Radiation Therapy Oncology Group (RTOG) 90-05 suggested that 15, 18, and 24 Gy could eradicate brain tumors of 31–40, 21–30, and less than 20 mm, respectively (45). Cardiac SBRT 25 Gy exhibited robust clinical efficacy in humans and shortened the QRS interval to electrophysiologic reprogramming (46). However, conduction velocity reprogramming could be achieved by treatment with as little as 15 Gy of radiation as well as 25 Gy does, suggesting that 15 Gy of RT may be a good dose to achieve electrical conduction reprogramming without transmural fibrosis (46). Interestingly, the RT dose that caused the formation of connective tissue in the myocardium of the left ventricle and septum significantly in the canine study by 2 Gy fractions was 68.0 Gy, and myocytolysis was 70.4 Gy given in 2 Gy fractions (13). Corrected for fractionation with alpha/beta ratio = 2.5, the EQD2 values of 15 and 18 Gy in one fraction were 58.3 and 82.0 Gy, respectively. In other words, conduction velocity reprogramming may be induced by 15 Gy, and 15 Gy does not cause myocardium fibrosis because its EQD2 is less than 68.0 Gy. The EQD2 of 18 Gy equal to 82.0 Gy (i.e., over 68 Gy) means it has a greater chance of causing transmural fibrosis. These lines of evidence support the potential strategy of giving 12 Gy of SBRT to the PTV, and the dose grading in the ITV and CTV to substate was 15 and 18 Gy, respectively. The results for the patient reported here were efficient, as confirmed by high-density mapping, MRI and EKG (Figures 2B,D,F).

Darby et al. (47) reported that rates of major coronary events increased linearly with the mean dose to the heart by 7.4% per gray. Hohmann et al. (48) noted that 3 months after VT was treated by SBRT, the left ventricular ejection fraction (LVEF) decline was correlated with mean dose and V20Gy. Considering the respective EQD2 with alpha/beta ratios = 10 and 2.5 for acute and late effect calculations, the mean heart dose was decreased by 6 Gy (71%) and 9 Gy (80%), respectively. On the other hand, the respective risk of acute and late toxicity of coronary events or heart toxicity could be reduced by 42 and 67%, respectively. Similarly, the acute and late effect doses to the heart substructures were decreased by 40–93 and 58–98%, respectively, when compared with treatment with 25 Gy. All together, the evidence supports the hypothesis that the administration of a lower radiosurgery dose to stop VT recycling and prevent damage to cardiac tissue in the long term may be a feasible idea, but more cases and longer follow-up times are needed to ensure efficacy. Additionally, the patient experienced recurrent VT, and a repeat bipolar voltage mapping study with the 3D CARTO mapping system confirmed a new lesion located at the RVOT, which was explained using the CARTO mapping system to check the electric reentry activity before retreatment. Because a lower radiosurgery dose provides a second chance to re-irradiate patients safely when they experience recurrent VT, relocalization of recurrent VT becomes more important for secondary irradiation.

To the best of our knowledge, this case report is the first in the world of a patient with refractory VT treated with SBRT using 12 Gy, and successful local treatment was confirmed by the 3D CARTO system, cardiac MRI and serial clinical EKG, suggesting that 12 Gy is a good dose for refractory VT. However, this study only describes the results from the first patient who was successfully treated with SBRT at this dose. Further study of more patients is warranted to assess the long-term efficacy and side effects of this treatment, as well as its mechanism of action and cost-effectiveness.

## Data availability statement

The original contributions presented in this study are included in the article/supplementary material, further inquiries can be directed to the corresponding author.

## Ethics statement

The studies involving human participants were reviewed and approved by the Human Experimentation Committee of Far Eastern Memorial Hospital (FEMH108074-F). The patients/participants provided their written informed consent to participate in this study. Written informed consent was obtained from the participant/s for the publication of this case report.

## Author contributions

C-HH, S-HH, Y-WW, and P-WS contributed to conception and design of the study. S-YW, M-CT, H-JT, and H-PY performed the image-fusion localization. Y-HL, W-PC, and H-HL take care of patient. C-HH and S-HH wrote the first draft of the manuscript. All authors contributed to manuscript revision, read, and approved the submitted version.
